# Five‐year analysis from phase 2 trial of “sandwich” chemoradiotherapy in newly diagnosed, stage IE to IIE, nasal type, extranodal natural killer/T‐cell lymphoma

**DOI:** 10.1002/cam4.569

**Published:** 2015-12-02

**Authors:** Li Zhang, Ming Jiang, Li Xie, Hong Zhang, Yu Jiang, Qun‐pei Yang, Wei‐ping Liu, Wen‐yan Zhang, Hong‐yu Zhuo, Ping Li, Nian‐yong Chen, Sha Zhao, Feng Wang, Li‐qun Zou

**Affiliations:** ^1^ Department of Medical OncologyState Key LaboratoryCancer CenterWest China Hospital of Sichuan UniversityChengduChina; ^2^ Department of OncologyDujiangyan Medical CenterDujiangyanSichuanChina; ^3^ Radiation Oncology of Cancer CenterWest China Hospital of Sichuan UniversityChengduChina; ^4^ Pathology DepartmentWest China Hospital of Sichuan UniversityChengduChina

**Keywords:** Chemoradiotherapy, extranodal natural killer/T‐cell lymphoma, long‐term survival, l‐asparaginase prednisone, prognostic factors, vincristine

## Abstract

The “sandwich” protocol, was first proposed by us and comprised of l‐asparaginase, vincristine, and prednisone chemotherapy with radiotherapy, results in 2‐year overall survival and progression‐free survival rates that surpass traditional therapies for patients with newly diagnosed, stage IE‐IIE, nasal type, extranodal natural killer/T‐cell lymphoma. The results had been published by cancer. These patients were followed up over a median period of 67 months, for which updates and the results of prognostic factors analyses are presented. The 5‐year overall survival and progress‐free survival rates were both 64%. The highest rates of death occurred during the first 6 months, and between the second and third year after enrollment. The initial therapeutic response (odds ratio = 5.83; *P *=* *0.001) and B symptoms (odds ratio = 6.13; *P* = 0.043) were significant prognostic factors for overall survival. However, the international prognostic index was not significant for progress‐free survival and overall survival. There were no severe long‐term side effects. These results indicate that the “sandwich” protocol may benefit the long‐term survival of patients with newly diagnosed stage IE‐IIE, nasal type, extranodal natural killer/T‐cell lymphoma. However, additional studies with larger samples are required to confirm these results. This study is registered at www.Chictr.org (ChicTR‐TNC‐09000394).

## Introduction

Extranodal natural killer (NK)/T‐cell lymphoma, nasal type (ENKTL) is a common subtype of lymphomas in East Asia and Latin America, accounting for 7–11% of lymphomas. Especially in China, it occupies the second place in lymphoma. Although two‐thirds of patients have early stage disease in the nasal cavity and its adjacent sites, the prognosis is poorer than for other types of early lymphoma. There is currently no standard first‐line therapy for newly diagnosed stage IE‐IIE ENKTL. Although a favorable overall response rate (ORR) is obtained with radiotherapy (RT) 1, 2, 3, 20–40% of patients experience relapse or progression during follow‐up 3, 4. ENKTL cells express elevated levels of p‐glycoprotein, which results in primary multidrug resistance (MDR) 5. As a result, traditional anthracycline‐based chemotherapies have a poor therapeutic outcome, even when combined with RT 1, 2, 6, 7, 8, 9, 10. Stage IE‐IIE patients with 0–1 Eastern Cooperative Oncology Group (ECOG) performance status show two‐ to three‐year 59–70% overall survival (OS) and 53–60% progression‐free survival (PFS) with anthracycline‐based chemotherapies 6, 11, 12, 13. In recent years, novel chemotherapeutic regimens, including non‐MDR‐dependent drugs combined with RT, have shown remarkable effects for stage IE‐IIE ENKTL, with 81–100% ORR, 73–82% complete remission (CR), 78–87% two‐ to three‐year OS, 33–97% grade 3–4 hematologic toxicity, and are accompanied only by common, and no fetal, side effects 14, 15, 16, 17. Some of these regimens are suggested by National Comprehensive Cancer Network Clinical Practice Guidelines (NCCN Guidelines) Non‐Hodgkin's lymphomas.

In a previous report 18, we firstly described an effective “sandwich” treatment therapy comprised of l‐asparaginase, vincristine, and prednisone (LVP) chemotherapy with RT. This method provided an 88.5% ORR, with a CR rate of 80.8%, and two‐year OS and PFS of 88.5 and 80.6%, respectively, which are higher than previous reports with other therapies, and is cited by NCCN Guidelines, Non‐Hodgkin's lymphomas. This article presents a follow‐up of these patients for a median of 67 months, along with 40 months of additional follow‐up data not previously presented. Specifically, we report OS, PFS, long‐term side effects, and prognostic factors.

## Methods

### Patients and procedures

The design and conduct of the study have been described in detail previously 18. Briefly, eligible patients included those with histologically proven ENKTL classified as Ann Arbor stage IE or IIE. Patients received chemotherapy every 3 weeks, consisting of l‐asparaginase (6000 IU/m^2^, iv) on days 1–5, vincristine (1.4 mg/m^2^, iv) on day 1, and prednisone (100 mg, oral) on days 1–5. After the second cycle of chemotherapy, RT with 56 Gy in 28 fractions, once a day, and five fractions every week was initiated. One week following completion of RT, chemotherapy was resumed for two to four cycles.

The final follow‐ups were performed in January 2015. Patients were required to go to the hospital for periodic review: every 3 months for the first 2 years, and every 6 months thereafter, for up to 5 years. Clinical investigations were the same as the baseline evaluations 18. This study is registered at www.Chictr.org and approved by the ethics committee (ChicTR‐TNC‐09000394).

### Data collection and endpoints

The primary endpoints were OS, defined as the time from enrollment to the end of follow‐up or death, and PFS, defined as the time from enrollment to the end of follow‐up or discovery of disease progression or relapse. Patients were monitored for factors influencing prognosis and long‐term side effects, including age, sex, therapeutic response, ECOG performance status, serum lactate dehydrogenase, B symptoms, regional lymph node, and Ann Arbor stage. Prognoses were indicated by the International Prognostic Index (IPI), which is used to evaluate diffuse large B‐cell lymphoma, and the Korea Natural killer/T‐cell Lymphoma Prognostic Index (NKIPI) 19.

### Statistical analysis

All analyses were performed using SPSS version 16.0 statistical software (SPSS Inc., Chicago, IL). Survival curves were drawn using the Kaplan–Meier method. Cox Proportional Hazards regression was used to adjust for various prognostic factors, and log‐rank tests were used to assess the prognostic value of IPI and NKIPI, and compare groups with and without CR. *P *≤* *0.05 was considered statistically significant.

## Results

### Five‐year survival events

**Figure 1:**
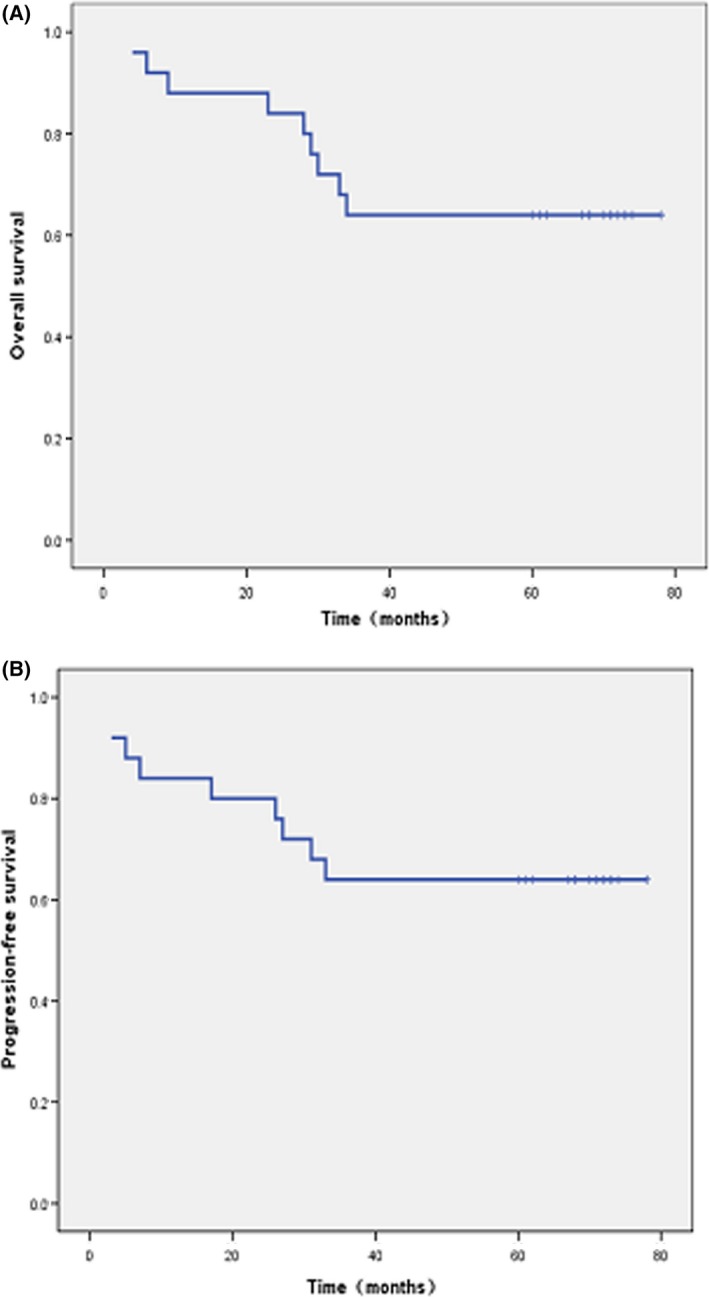
Survivals of patients with newly diagnosed, stage IE to IIE ENKTCL treated with the “sandwich” regimen. (A) Overall survival for the whole cohort. (B) progression‐free survival for the whole cohort.

**Figure 2:**
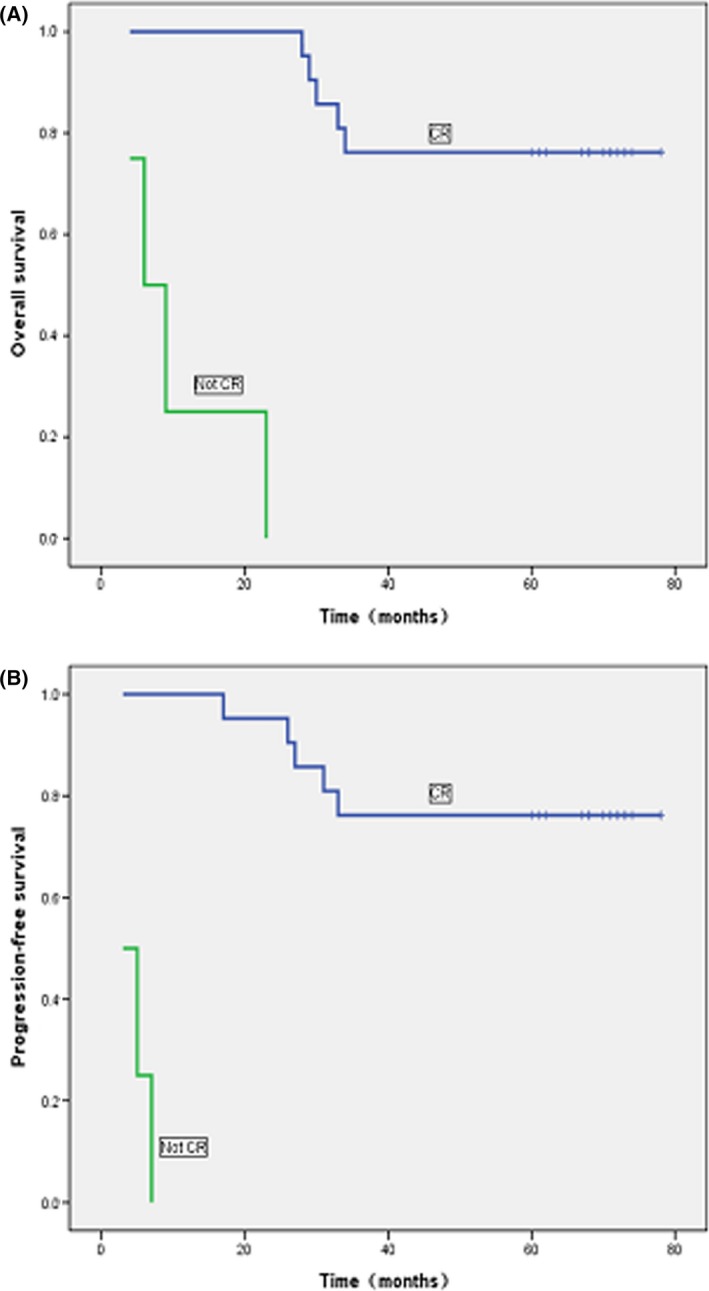
Impact of initial therapeutic response on survivals of patients with newly diagnosed, stage IE to IIE ENKTL. (A) Significant impact of complete response on overall survival. (B) Significant impact of complete response on progression‐free survival.

Patient characteristics and treatment outcomes have previously been described in detail 18. Briefly, a total of 26 patients (median age 43.5 years) were enrolled in the study from July 2008 to November 2009. One patient was lost during the initial 25‐month follow‐up and was excluded from analyses. The median follow‐up period for this study was 67 months (range: 4–78 months). Five patients experienced fatal disease progression, including two with skin metastases who died after 23 and 26 months of follow‐up, two with bone marrow infiltration that died after 27 and 31 months, and one patient with intestinal metastasis that died after 33 months. These patients had received various second‐line therapies: gemcitabine, cisplatin, and dexamethasone; gemcitabine, oxaliplatin, and l‐asparaginase; or methotrexate, ifosfamide, dexamethasone, etoposide, and l‐asparaginase. The 5‐year cumulative incidences of systemic and local failures were 30.8 and 3.8%, respectively. The highest rates of death occurred during the first 6 months, and between the second and third year after enrollment (Table 1). The 5‐year PFS and OS were both 64% (Fig. 1). Subgroup analysis showed that the 5‐year OS rate was maintained at 76% in patients with CR, and 0% in non‐CR patients (Fig. 2).

**Table 1:** Five‐year cumulative failure events

First region of progression	Cases (%)	Time to progression/Time to death
Bone marrow	4 (15.4)	3 months/4 months
		5 months/6 months
		27 months/30 months
		3 months/33 months
Skin	3 (11.5)	3 months/23 months
		26 months/28 months
		17 months/29 months
Intestinal	1 (3.8)	33 months/34 months
Oropharynx	1 (3.8)	7 months/9 months

### Prognostic factors

Complete remission was identified as a significant prognostic factor for PFS (*χ* ^2^ = 33.086; *P *=* *0.000) and OS (*χ* ^2^ = 35.015; *P *=* *0.000). Cox proportional hazards regression for OS showed that initial therapeutic response and B symptoms were also significant prognostic factors (*P *<* *0.05; Table 2). The probability of long‐term survival in patients who achieved CR was 5.8 times higher than those with partial response and 43 times higher those with progressive disease. Similarly, the absence of B symptoms increased the probability of OS, but not PFS, to 6.1 times that of patients with symptoms. The quality of therapeutic response for PFS did not meet the conditions of Cox proportional hazards regression. Log‐rank analyses for OS and PFS showed that IPI and NKIPI had no significant prognostic value (Table 2).

**Table 2:** Analysis of prognostic factors

	Patients (%)	PFS	OS	
		*P* values	*P* values	OR values
Age, average (range), years^a^	43.36 (18–74)	0.888	0.931	/
Sex^a^				
Male	72	0.400	0.336	/
Female	28			
Response^a^				
CR	84	b	0.001	5.833
PR	4			
PD	12			
ECOG performance status^a^				
0	60	0.400	0.135	/
1	36			
2	4			
Serum LDH^a^				
Normal	60	0.458	0.091	/
Increased	40			
Regional nodes^a^				
No	76	0.159	0.122	/
Yes	24			
B symptoms^a^				
Absent	64	0.244	0.043	6.130
Present	36			
Ann Arbor stage^a^				
Limited I E	52	0.149	0.206	/
Extensive I E^c^	24			
II E	24			
IPI scores^d^				
0	48	0.768	0.722	/
1	44			
2	8			
NKIPI scores^d^				
0	36	0.178	0.164	/
1	32			
2	24			
3	8			

OS, Overall survival; PFS, Progression‐free survival; CR, complete response; PR, partial response; PD, progressive disease; ECOG, Eastern Cooperative Oncology Group; LDH, lactate dehydrogenase; IPI: International Prognostic Index; NKIPI, Korea natural killer/T‐cell lymphoma Prognostic Index.

a Cox regression.

b Inadequate for statistical power.

c Tumors extended beyond the nasal cavity and into the neighboring structures without any sign of nodal or distant dissemination.

d Log‐rank test.

### Long‐term side effects

Long‐term side effects were infrequent, with nasal mucosa drying and bleeding occurring in 8% (2/25) of patients. There were no incidences of neurotoxicity, bone marrow suppression, organ dysfunction, or secondary tumors reported.

## Discussion

The standard therapy has not yet been established for ENKTL. CHOP and CHOP‐like chemotherapy show poor antitumor activity against ENKTL, and even combined with RT, which do not make patients getting survival benefit. In a retrospective study of 79 stage I‐IIE patients with ENKTL, Cheung et al. 1 found no difference in 5‐year survivals (36–38%) among patients who received RT in addition to chemotherapy with cyclophosphamide and hydroxydaunorubicin with vincristine and prednisone. Similar 5‐year survivals were reported in another study of 36 patients with newly diagnosed ENKTL 20. Although 5‐year survivals of >70% have been reported with anthracycline‐based chemotherapy combined with involved‐field RT, the induction of chemotherapy in these studies did not afford survival benefits 2, 10. Therefore, the protocol of anthracycline‐based chemotherapy combined RT do not make patients getting survival benefit.

Current data indicated that protocols employing non‐MDR‐dependent drugs together with RT produced favorable ORR, CR rates, and 2‐ to 3‐year OS and PFS (Table 3), including our first report 18. According to the above results, concurrent chemoradiotherapy (CCRT) and sequential chemoradiotherapy (SCRT) are suggested by NCCN Guidelines. However, long‐term follow‐up studies on survival were rarely published. Up to now, there are two articles reporting the 5‐year OS and PFS. Kim et al. conducted a phase II trial of CCRT followed by 2 cycles of l‐asparaginase‐containing chemotherapy for patients who were newly diagnosed with stages IE and IIE ENKTL, 5‐year PFS and OS were 60 and 73%, respectively 21. Yamaguchi et al. reported the updated results of CCRT that consisted of 50 Gy of RT and three cycles of dexamethasone, etoposide, ifosfamide, and carboplatin(DeVIC), OS at 5 years was 70%, and the PFS at 5 years was 63 22. The “sandwich” regimen of LVP chemotherapy with radiotherapy, which was firstly proposed by us, produced a significant short‐term curative rate with generally well tolerated by patients. Now the updated results were showed, the median follow‐up time of 67 months (range, 4–78 months), 5‐year OS, and PFS were 64%, and subgroup analysis according to quality of response showed that 5‐year OS rates was 76% for patients in CR versus 0% for those with non‐CR (Figs. 1 and 2). Our results indicate that the “sandwich” protocol produced effect as well as regimens suggested by NCCN guidelines.

**Table 3:** Prognostic factors analyzed in articles

Reference number	14	15	16	17	18
OS (%)	3y (86.3)	2y (78)	2y (80.1)	2y (86)	2y (88.5)
PFS (%)	3y (85.2)	2y (67)	2y (81)	2y (86)	2y (80.6)
Age (%)					
Less than or equal to 60 years	86.7	74	94.7	85.2	84.6
Older than 60 years	13.3	26	5.3	14.8	15.4
Sex (%)					
Male	56.7	63	63.2	59.3	73
Female	43.3	37	36.8	40.7	27
ECOG performance status (%)					
0	56.7	78	34.2^a^	18.5	57.7
1	43.3	15	63.2	66.7	38.5
2	/	7	2.6	14.8	3.8
Ann Arbor stage (%)					
I	50	67	52.6^a^	66.7	76.9
II	50	33	28.9	33.3	23.1
III/IV	/	/	18.4	/	/
LDH (%)					
Normal	80	81	81.4^a^	88.9	61.5
Increased	20	19	18.6	11.1	38.5
B symptoms (%)					
Present	36.7	37	57.9^a^	37	34.6
Absent	63.3	63	42.1	63	65.4
EBV titration (%)					
At least 64 copies/*μ*L	36.7^a^	/	/	/	/
Lower than 64 copies/*μ*L	63.3	/	/	/	/
Regional node (%)					
Yes	36.7	/	47.4	40.7	/
No	63.3	/	52.6	59.3	/
IPI scores (%)					
0	76.7 (0–1)	59	71.1 (0–1)^a^	81.5	50
1		30		18.5	42.3
2	23.3	11	18.4	/	7.7
3	/	/	5.3	/	/
4‐5	/	/	5.3	/	/
NKIPI scores (%)					
0		/	26.3^a^	/	
1	70 (1–2)	33	34.2	/	69 (1–2)
2		30	18.4	/	
3	30 (3–4)	26	21.1 (3–4)	/	31 (3–4)
4		11		/	
Platelet count (%)					
Lower than or equal to 150 000/mm^3^	10^a^	/	/	/	/
Higher than 150 000/mm^3^	90	/	/	/	/
Lymphocyte count (%)					
Lower than or equal to 1000/mm^3^	23.3^a^	/	/	/	/
Higher than 1000/mm^3^	76.7	/	/	/	/
CR^a^ (%)	80	77	81.6	74.1	80.8
ORR (%)	83.3	81	84.2	96.3	88.5

OS, Overall survival; PFS, Progression‐free survival; CR, complete response; ORR, overall response rates; ECOG, Eastern Cooperative Oncology Group; EBV, Epstein–Barr virus; LDH, lactate dehydrogenase; IPI: International Prognostic Index; NKIPI, Korea natural killer/T‐cell lymphoma Prognostic Index.

a Prognostic factors.

Compared with other protocols 14, 15, 16, 17, 21, grade 3 to 4 short‐term toxicity were infrequent in “sandwich” protocol. Long‐term toxicities were rarely published. Yamaguchi et al. 22 reported that one patient treated with RT‐2/3DeVIC experienced perforation of the nasal skin as a grade 4 late RT adverse event, one patient treated with RT‐100%DeVIC experienced grade 3 irregular menstruation, eleven patients (33%) experienced grade 1 or 2 late RT adverse events of the eye. By contrast, the “sandwich” treatment therapy generated long‐term toxicities which were infrequent and not serious, only two patients (8%) experienced nasal mucosa drying and bleeding occurrence. So the long‐term side effects in “sandwich” regimen were milder.

Interestingly, ECOG performance status, Ann Arbor stage, B symptoms, paranasal extension, and perforation have been identified as significant prognostic factors for anthracycline‐based regimens prognosis 1, 2, 6, 10, 12, and Epstein–Barr virus titration, platelet and lymphocyte counts, ECOG performance status, Ann Arbor stage, lactate dehydrogenase, and B symptoms were prognostic for non‐MDR‐dependent drugs regimens (Table 3). Our present analysis identified only treatment response and B symptoms as significant factors for OS. The presence of discrepancies among these results indicates that a unified study is needed that includes multiple centers from several countries. A consistent finding, however, concerns the improved survival of patients with CR, indicating that optimal first‐line treatments are crucial. In addition, multiple studies, including the present report, indicate that IPI and NKIPI are not significant predictors of long‐term survival (Table 3). Other studies have evaluated additional prognostic models, but with varying results 19, 23, 24, 25. Further investigations are needed to determine the universality and applicability of these models.

In conclusion, the results of our study demonstrate that the “sandwich” protocol comprised of l‐asparaginase‐based chemotherapy with RT and consolidated chemotherapy may provide a long‐term survival advantage with minimal side effects in newly diagnostic stage IE‐IIE ENKTL patients. The findings also confirm that the treatment response significantly affects prognosis. Furthermore, we found that IPI and Ann Arbor staging, which are universally used in non‐Hodgkin's lymphoma, are limited for predicting prognoses in patients with ENKTCL. As the sample sizes in this study are small and there was no control group, further controlled trials with larger cohorts are needed to verify the superiority of the “sandwich” protocol and explore a suitable staging system and IPI.

## Conflict of Interest

None declared.
